# Physical and Psychosocial Well-Being of Hospitalized and Non-Hospitalized Patients With COVID-19 Compared to the General Population in Qatar

**DOI:** 10.3389/fpsyt.2021.792058

**Published:** 2021-12-13

**Authors:** Sami Ouanes, Hassen Al-Amin, Nurrunnazha Binti Hussein, Faisal Khan, Ahmad Al Shahrani, Premalatha David, Amel Baker Wali, Maliha Thapur, Mustafa Abdul Karim, Muna Al Maslamani, Zainab Al-Ansari, Suhaila Ghuloum

**Affiliations:** ^1^Department of Psychiatry, Hamad Medical Corporation, Doha, Qatar; ^2^Weill Cornell Medicine, Psychiatry Department, Doha, Qatar; ^3^Communicable Disease Center, Hamad Medical Corporation, Doha, Qatar; ^4^Weill Cornell Medicine, Medical Education, Doha, Qatar

**Keywords:** COVID-19, functioning, quality of life, hospitalized, quarantined, general population

## Abstract

**Background:** Many studies have shown a high prevalence of depression, anxiety, and stress symptoms in COVID-19 patients and the general population. However, very few studies directly examined the potential impact on the health-related quality of life (HRQoL), and none compared HRQoL in COVID-19 patients to the general population amid the pandemic.

**Methods:** We carried out a cross-sectional study comparing HRQoL (as measured using the RAND Short Form 36 or SF-36 Health Survey) in randomly selected individuals from three different groups: hospitalized COVID-19 patients, quarantined COVID-19 patients, and controls from the general population in Qatar. We constructed a multivariate analysis of covariance (MANCOVA) to compare the SF-36 scores between the three groups and control for various covariates.

**Results:** Our sample consisted of 141 COVID-19 inpatients, 99 COVID-19 quarantined patients, and 285 healthy controls. Surprisingly, we found that HRQoL was higher in COVID-19 hospitalized than in COVID-19 non-hospitalized patients than in controls. The main components where COVID-patients scored higher than controls were physical functioning and role limitations due to emotional problems. In COVID-19 patients, the female gender, older age, and past psychiatric history were associated with lower HRQoL.

**Conclusions:** It seems that COVID-19 patient's HRQoL might be better than expected. Our results can be explained by social support from family and friends, easy access to mental health screening and care, and a possible change of perspectives after recovery from COVID-19, resulting in psychological growth and enhanced resilience.

## Introduction

Amidst the Coronavirus disease (COVID-19) pandemic, more reports showed an increase in the prevalence of depressive, stress, and anxiety symptoms in the general population. Indeed, a meta-analysis of the global prevalence of mental health problems among the general population during the COVID-19 pandemic found a prevalence of 28.0% (95% CI = 25.0–31.2%) for depression, 26.9% (95% CI = 24.0–30.0%) for anxiety, and 36.5% (95% CI 30.0–43.3%) for stress symptoms (1).

A meta-analysis of the prevalence of depression, anxiety, and insomnia symptoms in patients with COVID-19 found a pooled prevalence of 38% (95% CI = 25–51%) for depression, 38% (95% CI = 24–52%) for anxiety, and 48% (95% CI = 11–85%) for insomnia (2). Similarly, a systematic review of psychiatric sequelae of COVID-19 highlighted high rates of depressive (10.0 and 68.5%), anxiety (5.0 and 55.2%), and acute stress reaction (10.0 to 28.0%) symptoms, as well as high rates of insomnia (26.0 to 52.2%). Even months after recovery, 7.0 to 36.4% of patients endorsed symptoms suggestive of post-traumatic stress disorder (PTSD), and 40.0 to 69.0% reported persistent fatigue 2 to 3 months after discharge, with a significant impact on their activities of daily living and quality of life (3). All of these symptoms can affect individual's physical and psychosocial well-being.

However, in stark contrast with the very large number of studies examining depressive, anxiety, and stress symptoms in COVID-19 patients, only a few studies directly examined the health-related quality of life (HRQoL). Some studies reported that HRQoL is low in COVID-19 patients 1–3 months after discharge (4, 5); others reported that COVID-19 patients were scoring lower than norms on certain components of the HRQo (6, 7).

One fundamental limitation of these studies is the lack of a control arm, and most simply used previously established norms to interpret COVID-19 patient's HRQoL scores (8). Thus, it is better to have a control group to discern the direct effects of COVID-19 infection on HRQoL from the overall impact of the pandemic on the whole population. Indeed, some studies reported high rates of depression, anxiety, and stress in the general population (1), and others found low HRQoL (9, 10) in the general population amidst the pandemic.

This study aimed to address some of the shortcomings of the previous studies by (i) directly examining the HRQoL amid the pandemic rather than assuming that depressive, anxiety, and stress symptoms translate into poorer HRQoL; (ii) comparing the HRQoL in COVID-19 patients to a sample from the general population group; (iii) comparing two different groups of COVID-19 patients (hospitalized vs. quarantined).

## Methods

We conducted this cross-sectional study in July-September 2020 in the State of Qatar to examine HRQoL in COVID-19 patients compared to a sample from the general population. We used hospitalization vs. non-hospitalization as a proxy for COVID-19 severity.

We included three groups:

- A group of individuals diagnosed with COVID-19 and admitted to hospital (inpatient group)
- A group of individuals diagnosed with COVID-19 but not requiring hospital admission (quarantine group)
- A general population sample (control group)

We enrolled participants through phone interviews or an anonymous online version of the same questionnaire through Survey Monkey. We used phone interviews for cases tested positive for COVID-19, hospitalized or quarantined. For the general population sample, we sent a link to the online version by phone.

### Participants

The inclusion criteria for the three groups were the following: adults aged 18–65 years who could speak Arabic or English. For the inpatient group, we excluded patients at the intensive care unit at the time of the study.

For COVID-19 groups (both inpatient and quarantine), we used the national records of COVID-19 hospitalized or quarantined patients to select the participants randomly. Then, we contacted every 10^th^ name inviting them to participate until we reached the required number.

For the three groups, we chose *not* to exclude subjects with past history of psychiatric disorders, because: (i) this would allow us to examine the HRQoL in this subpopulation as well, and to examine the effect of past psychiatric history as a potential predictor for the HRQoL in COVID-19 patients vs. controls (ii) using MANCOVA, we can control for the effect of the presence of past history of psychiatric disorders in the comparison between groups.

We allocated the randomly chosen cases to members of the research team, who then contacted the patients by phone. The interviewers were blinded to whether the patients were hospitalized or quarantined to avoid potential bias.

The research team member explained the purpose of the phone call and invited the patient to participate in the study. After granting the consent, we offered participants to conduct the interview either over the phone or using a web-based survey.

We sent text messages through a mobile service operator to 10,000 subjects, randomly selected English and Arabic speakers to recruit the general public group. The text messages contained a brief explanation of the study and a link to the anonymous online survey.

#### Sample Size Calculation

We calculated the sample size using the one-way ANOVA to compare three means (scores of SF-36 for each group) followed by pairwise comparisons with two-sided equality. Thus, we had three groups (i.e., three pairwise comparisons). In addition, we wanted a significant difference of 33% for each comparison, with a standard deviation of 10, a power of 80%, and a type one error of 5% (significance level). Based on these parameters, the calculated sample size was 83 subjects for each group. Further, we elected to recruit at least 100 subjects for each group to account for 20% of dropouts or incomplete data.

### Measurements

The survey consisted of questions about basic sociodemographic information and sleep and an assessment of the HRQoL using the RAND Short Form 36 (SF-36) Health Survey in English or Arabic (depending on the participant's preferred language).

The RAND SF-36 questionnaire is a widely used 36-item questionnaire to evaluate HRQoL. It covers eight domains of physical and mental well-being: physical functioning, role limitations due to physical health, role limitations due to emotional problems, energy/fatigue, emotional well-being, social functioning, pain, and general health (11).

We interpreted and analyzed the SF-36 scale results using the scoring rules detailed on the RAND corporation Website (RAND Corporation, 12). For each scale, the score ranges from 0 to 100, with 100 indicating the best health. These eight concepts have also been summarized into two scales: a physical component score and a mental component score (13). The questionnaire was constructed for self-administration and by telephone interview (14). The English version of the SF-36 has demonstrated excellent reliability (Cronbach's alpha for the eight scales ranging from 0.85 to 0.94) and discriminant validity (15). The Arabic version was previously validated and showed good reliability (median Cronbach's alphas exceeded 0.70 for every scale except for general health, which had an alpha value of 0.6), high correlations with the English version (ranging from 0.73 to 0.92), as well as good test-retest reliability (16). Previous studies used the Arabic SF-36 in different Arab countries, including Saudi Arabia (16), Jordan (17), Egypt (18), Tunisia (19), Lebanon (20), and Qatar (21).

#### Ethical Considerations

Hamad Medical Corporation Institutional Review Board (MRC-05-045) approved this study.

Informed consent was obtained from all participants. All the research team members were bilingual (Arabic and English) and received similar training to standardize the consent process. Participants were offered a consultation with mental health services if needed.

### Statistical Analysis

Data were analyzed using SPSS for Windows, version 26.

#### Descriptive Statistics

We determined absolute and relative frequencies for categorical variables and the mean and standard deviation (SD) for the continuous ones.

#### Analytical Statistics

To compare categorical variables between groups (inpatient group, quarantine group, and controls), we used Pearson's Chi-square and, in case of non-validity (cells with an expected count <5), Fischer's exact test. To compare continuous variables between groups (pairwise), we used the *t*-test for independent samples.

We chose to use the APA style in the table reporting chi-square and *t*-test results because the high number of comparisons would otherwise make the table too complex, and difficult to read.

We used a one-way analysis of variance to examine the associations between the SF-36 physical and mental component scores and the categorical variables. For the variables that did not follow a normal distribution (as per the Shapiro-Wilk test), we used non-parametric correlations to examine the associations between the SF-36 physical and mental component scores and the continuous sociodemographic and clinical variables.

To compare the SF-36 scale scores between the three groups (inpatient, quarantine, and controls), we constructed a multivariate analysis of covariance (MANCOVA): the SF-36 scores (physical functioning, role limitations due to physical health, role limitations due to emotional problems, energy/fatigue, emotional well-being, social functioning, pain, general health) as dependent variables; and the group (inpatient vs. quarantine vs. controls) as a fixed factor, with gender, age, nationality group (dichotomized as belonging to the most represented group among COVID-19 patients, i.e., Indian Subcontinent vs. others), education (dichotomized as higher education vs. others), occupation (dichotomized as belonging to the most represented group among COVID-19 patients, i.e., craft and manual workers vs. others), and previous psychiatric history as covariables. We chose to conduct a MANOVA rather than a multiple regression analysis because the SF-36 has eight components that cannot be summarized into one total score (12). Preliminary assumptions for MANCOVA (including normality, linearity, univariate and multivariate outliers, covariance matrices, and multicollinearity) were tested. Pillai's trace test was used because the SF-36 scores violated the normality assumption. The effect size was assessed using the partial eta squared. The alpha value was set at 0.05. Finally, we adjusted *p* values for multiple comparisons using Bonferroni's method.

## Results

### Sociodemographic and Clinical Characteristics

Our sample consisted of 141 inpatients, 99 quarantined individuals with COVID-19, and 285 controls (subjects from the general population who were neither infected nor quarantined) (Table 1).

**Table 1:** Sociodemographic and clinical features of COVID-19 inpatients vs. COVID-19 infected quarantined individuals vs. controls.

		**A: Inpatient *n* = 141**	**B: Quarantine *n* = 99**	**C: Controls *n* = 285**
Gender, Male, *n* (%)		121 (85.8%)_a_	69 (69.7%)_b_	122 (42.8%)_c_
Age, in years, m ± SD		44.0 ± 10.5_a_	36.8 ± 10.4_b_	38.6 ± 10.2_b_
Nationality Group, *n* (%)	Qatar	21 (14.9%)_a_	3 (3.0%)_b_	30 (10.5%)_a, b_
	Arab countries other than Qatar	16 (11.3%)_a_	32 (32.3%)_b_	37 (13.0%)_a_
	Indian Subcontinent	90 (63.8%)_a_	58 (58.6%)_a_	6 (2.1%)_b_
	Southeast Asia	8 (5.7%)_a_	4 (4.0%)_a_	127 (44.6%)_b_
	Other	6 (4.3%)_a_	2 (2.0%)_a_	85 (29.8%)_b_
Occupation, *n* (%)	Unemployed	1 (0.7%)_a_	3 (3.0%)_a, b_	17 (6.0%)_b_
	Housewife	6 (4.3%)_a_	12 (12.1%)_a_	32 (11.2%)_a_
	Craft and Manual Worker	83 (58.9%)_a_	26 (26.3%)_b_	8 (2.8%)_c_
	Professional	45 (31.9%)_a_	53 (53.5%)_b_	210 (73.7%)_c_
	Student	3 (2.1%)_a_	5 (5.1%)_a_	17 (6.0%)_a_
	Retired	3 (2.1%)_a_	0 (0.0%)	1 (0.4%)_a_
Education, *n* (%)	Primary or Middle School	54 (38.3%)_a_	14 (14.1%)_b_	4 (1.4%)_c_
	Secondary School	49 (34.8%)_a_	31 (31.3%)_a_	46 (16.1%)_b_
	Higher education	38 (27.0%)_a_	54 (54.5%)_b_	235 (82.5%)_c_
Past psychiatric history, yes, *n* (%)		5 (3.5%)_a_	3 (3.0%)_a_	13 (4.6%)_a_
Number of hours of sleep, m ± SD		7.1 ± 1.4_a_	7.2 ± 1.4_a_	7.1 ± 1.3_a_
SF-36 scale scores, m ± SD	Physical functioning	91.0 ± 15.1_a_	91.2 ± 14.7_a_	79.6 ± 25.3_b_
	Role limitations due to physical health	79.4 ± 35.9_a_	76.3 ± 38.4_a_	78.4 ± 32.3_a_
	Role limitations due to emotional problems	83.9 ± 34.9_a_	80.1 ± 36.2_a_	68.0 ± 39.5_b_
	Energy/fatigue	64.3 ± 21.8_a_	58.6 ± 22.7_a_	60.7 ± 20.5_a_
	Emotional well-being	77.7 ± 16.8_a_	75.2 ± 18.9_a, b_	71.0 ± 20.9_b_
	Social functioning	74.5 ± 26.6_a_	72.3 ± 28.9_a, b_	67.6 ± 26.8_b_
	Pain	83.9 ± 22.3_a_	83.1 ± 23.5_a_	83.2 ± 20.0_a_
	General health	77.6 ± 18.7_a_	78.9 ± 17.0_a_	74.9 ± 17.0_a_
SF-36 component scores, m ± SD	Physical component score	58.1 ± 6.6_a_	58.2 ± 6.3_a_	57.2 ± 7.0_a_
	Mental component score	51.2 ± 8.3_a_	49.5 ± 8.7_a, b_	47.9 ± 10.4_b_

*M, mean; SD, standard deviation; SF-36, 36-Item Short Form Health Survey. Comparisons are displayed using the APA style: values in the same row and subtable not sharing the same subscript (corresponding to each group in the first row) are significantly different at p < 0.05. Bonferroni correction was applied to multiple comparisons*.

The proportion of males was significantly higher in inpatients than in the quarantine sample than in controls. Mean age was also higher in inpatients than in quarantined individuals or controls. More craft and manual workers in the inpatient group than in quarantine than in controls. The inpatient group also had lower education levels than both other groups.

The percentage of participants who reported positive psychiatric history (anxiety, depression, or both) was comparable between groups (ranging between 3 and 4.6%). The number of sleep h was comparable across groups, with a mean of 7.1–7.2 h, and an SD of 1.3–1.4.

When comparing SF-36 scale scores, we found no significant differences between the inpatient and quarantine groups. However, controls reported lower physical functioning scores than inpatients and quarantined individuals. Similarly, controls scored worse on role limitations due to emotional problems than the inpatients or the quarantined groups. In emotional well-being and social functioning, controls scored lower than inpatients, but their scores did not significantly differ from quarantined subjects. In other subdomains (role limitations due to physical health, energy/fatigue, pain, and general health), controls did not differ significantly from either of the other groups.

The SF-36 physical component score did not differ between the three groups. Nevertheless, the mental component score was significantly lower in the control group than in the inpatient group. On the other hand, the mental component score in the quarantine group did not differ significantly from either of the other groups.

### Factors Associated With SF-36 Component Scores in Patients With COVID-19 (Either Inpatient or Quarantined)

The SF-36 physical component score was significantly lower in females, and was significantly associated with the nationality group and the occupation, but did not with education level or with past psychiatric history. We also did not find any correlation between the physical component score and age or the reported number of sleep hours (Table 2).

**Table 2:** Factors associated with SF-36 component scores in patients with COVID-19 (either inpatient or quarantined).

		**Mental component score**	**F**	***p*-value**	**Physical component score**	**F**	***p*-value**
Gender	Female	48.6 ± 9.2	3.128	0.078	55.6 ± 9.1	10.263	0.002
	Male	51.0 ± 8.2			58.8 ± 5.4		
Nationality Group	Qatar	48.8 ± 11.8	0.557	0.694	53.5 ± 10.1	3.914	0.004
	Arab countries other than Qatar	49.9 ± 8.0			58.6 ± 5.1		
	Indian subcontinent	50.7 ± 8.3			58.7 ± 6.1		
	Southeast Asia	52.4 ± 5.8			57.6 ± 4.8		
	Other	52.3 ± 7.4			60.6 ± 3.3		
Occupation	Unemployed	50.7 ± 8.8	1.386	0.230	56.8 ± 7.1	4.725	0.000
	Housewife	48.8 ± 10.1			59.2 ± 6.9		
	Craft and Manual worker	51.1 ± 7.9			58.4 ± 6.6		
	Professional	50.3 ± 8.2			58.0 ± 5.9		
	Student	53.3 ± 9.8			58.6 ± 6.2		
Education	Primary or Middle School	49.8 ± 9.4	0.415	0.661	58.0 ± 7.0	0.090	0.914
	Secondary school	51.1 ± 7.9			58.4 ± 6.6		
	Higher education	50.5 ± 8.3			58.0 ± 6.4		
Past psychiatric history	No	50.8 ± 8.5	6.479	0.012	58.3 ± 6.4	1.617	0.205
	Yes	43.1 ± 5.2			55.3 ± 5.9		

*SF-36, 36-Item Short Form Health Survey. SF-36 component scores are shown as mean ± standard deviation*.

The SF-36 mental component score was significantly lower in participants with past psychiatric history and was positively correlated with the reported number of sleep hours (Rho = 0.138, *p* = 0.033). However, we did not find any association between the SF-36 mental component score and gender, age, nationality group, or education level.

Further, the physical and the mental component scores were not significantly correlated (Rho = 0.062, *p* = 0.342).

### Multivariate Analysis

We used the MANCOVA analysis to compare the SF-36 scores in the three groups controlling for gender, age, past psychiatric history, education, nationality, and occupation. The results (Table 3) showed significant small effects of gender, age, and group (inpatient, quarantine, or controls. Past psychiatric history displayed significant effects on SF-36 scores, whereas education, nationality, and occupation did not show any significant effects.

**Table 3:** Multivariate covariance analysis comparing SF-36 scale scores between COVID-19 inpatients vs. COVID-19 infected quarantined individuals vs. controls controlling for sociodemographic and clinical variables.

**Effect**	**Pillai's Trace**	**F**	***p*-value**	**Partial Eta squared**
Gender	**0.053**	**3.567**	**0.000**	**0.053**
Age	**0.031**	**2.045**	**0.040**	**0.031**
Past psychiatric history	**0.079**	**5.448**	**0.000**	**0.079**
Education	0.021	1.355	0.214	0.021
Nationality	0.018	1.142	0.333	0.018
Occupation	0.011	0.736	0.659	0.011
Group	**0.094**	**3.160**	**0.000**	**0.047**

*SF-36, 36-Item Short Form Health Survey. Bold values indicate statistically significant results*.

**Figure 1:**
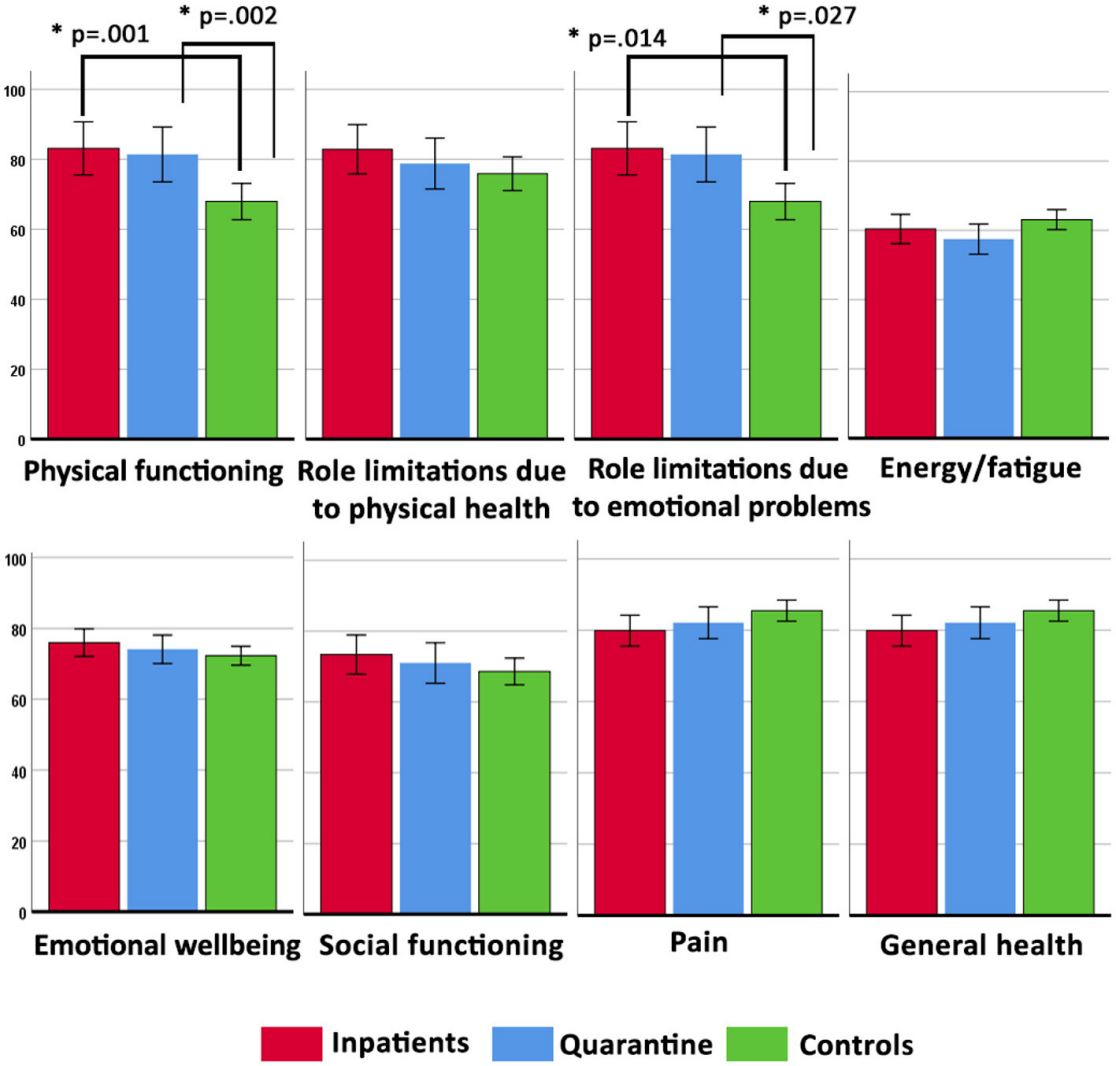
Estimated marginal means (with their 95% error bars) of the SF-36 scores in the inpatient, quarantine, and control groups, controlling for gender, age, past psychiatric history, education, nationality, and occupation. All *p* values were adjusted for multiple comparisons using the Bonferroni method.

Univariate tests of between-subjects' effects (Table 4) showed that group had a small effect on physical functioning and role limitations due to emotional problems. Age showed a significant small effect on physical functioning, whereas past psychiatric history had small effects on all SF-36 scale scores except physical functioning. Gender showed small effects on the following scale scores: role limitations due to emotional problems, energy/fatigue, emotional well-being, pain, and general health (Figure 1).

**Table 4:** Univariate tests of between-subject's effects with SF-36 scale scores as dependent variables, group (inpatients vs. COVID-19 infected quarantined individuals vs. controls) as a fixed factor, and sociodemographic and clinical characteristics as covariables.

	**Dependent Variable**	**Type III Sum of Squares**	**Mean Square**	**F**	***p*-value**	**Partial Eta Squared**
Gender	Physical functioning	419.757	419.757	0.945	0.331	0.002
	Role limitations due to physical health	741.223	741.223	0.634	0.426	0.001
	**Role limitations due to emotional problems**	**17599.406**	**17599.406**	**13.018**	**0.000**	**0.025**
	**Energy/fatigue**	**7243.418**	**7243.418**	**17.260**	**0.000**	**0.032**
	**Emotional well-being**	**2715.770**	**2715.770**	**7.646**	**0.006**	**0.015**
	Social functioning	2437.598	2437.598	3.380	0.067	0.007
	**Pain**	**3887.007**	**3887.007**	**8.857**	**0.003**	**0.017**
	**General health**	**2536.761**	**2536.761**	**9.059**	**0.003**	**0.017**
Age	**Physical functioning**	**3129.109**	**3129.109**	**7.046**	**0.008**	**0.013**
	**Role limitations due to physical health**	**1958.734**	**1958.734**	**1.674**	**0.196**	**0.003**
	**Role limitations due to emotional problems**	**4557.089**	**4557.089**	**3.371**	**0.067**	**0.006**
	**Energy/fatigue**	**782.612**	**782.612**	**1.865**	**0.173**	**0.004**
	**Emotional well-being**	**122.332**	**122.332**	**0.344**	**0.558**	**0.001**
	**Social functioning**	**1.251**	**1.251**	**0.002**	**0.967**	**0.000**
	**Pain**	**258.653**	**258.653**	**0.589**	**0.443**	**0.001**
	**General health**	**14.175**	**14.175**	**0.051**	**0.822**	**0.000**
Past psychiatric history	Physical functioning	1314.742	1314.742	2.961	0.086	0.006
	Role limitations due to physical health	6587.292	6587.292	5.630	0.018	0.011
	Role limitations due to emotional problems	14181.776	14181.776	10.490	0.001	0.020
	Energy/fatigue	7656.771	7656.771	18.244	0.000	0.034
	Emotional well-being	11106.885	11106.885	31.272	0.000	0.057
	Social functioning	8673.863	8673.863	12.028	0.001	0.023
	Pain	2205.210	2205.210	5.025	0.025	0.010
	General health	7609.925	7609.925	27.176	0.000	0.050
Education	Physical functioning	1012.351	1012.351	2.280	0.132	0.004
	Role limitations due to physical health	259.209	259.209	0.222	0.638	0.000
	Role limitations due to emotional problems	25.336	25.336	0.019	0.891	0.000
	Energy/fatigue	143.529	143.529	0.342	0.559	0.001
	Emotional well-being	0.024	0.024	0.000	0.993	0.000
	Social functioning	287.095	287.095	0.398	0.528	0.001
	Pain	642.052	642.052	1.463	0.227	0.003
	General health	943.465	943.465	3.369	0.067	0.006
Nationality group	Physical functioning	43.374	43.374	0.098	0.755	0.000
	Role limitations due to physical health	2621.681	2621.681	2.241	0.135	0.004
	Role limitations due to emotional problems	2098.261	2098.261	1.552	0.213	0.003
	Energy/fatigue	65.496	65.496	0.156	0.693	0.000
	Emotional well-being	163.234	163.234	0.460	0.498	0.001
	Social functioning	303.236	303.236	0.421	0.517	0.001
	Pain	5.768	5.768	0.013	0.909	0.000
	General health	1027.364	1027.364	3.669	0.056	0.007
Occupation	Physical functioning	354.009	354.009	0.797	0.372	0.002
	Role limitations due to physical health	1189.128	1189.128	1.016	0.314	0.002
	Role limitations due to emotional problems	1434.500	1434.500	1.061	0.303	0.002
	Energy/fatigue	11.276	11.276	0.027	0.870	0.000
	Emotional well-being	100.268	100.268	0.282	0.595	0.001
	Social functioning	1057.067	1057.067	1.466	0.227	0.003
	Pain	209.782	209.782	0.478	0.490	0.001
	General health	104.485	104.485	0.373	0.542	0.001
Group	**Physical functioning**	**7829.189**	**3914.595**	**8.815**	**0.000**	**0.033**
	Role limitations due to physical health	2339.098	1169.549	1.000	0.369	0.004
	**Role limitations due to emotional problems**	**13550.684**	**6775.342**	**5.012**	**0.007**	**0.019**
	Energy/fatigue	1638.254	819.127	1.952	0.143	0.008
	Emotional well-being	646.024	323.012	0.909	0.403	0.004
	Social functioning	1078.017	539.008	0.747	0.474	0.003
	Pain	1527.752	763.876	1.741	0.176	0.007
	General health	195.708	97.854	0.349	0.705	0.001

*Bold values indicate statistically significant results*.

## Discussion

In this cross-sectional study, we examined the physical and psychosocial well-being (or HRQoL as measured by the SF-36) of hospitalized and non-hospitalized patients with COVID-19 compared to the general population in Qatar. Surprisingly, we found that HRQoL was higher in COVID-19 hospitalized than in COVID-19 non-hospitalized patients. The latter also had higher HRQoL than controls. In COVID-19 (both hospitalized and non-hospitalized) patients, the main functioning components that were better than controls consisted of physical functioning and role limitations due to emotional problems. Among COVID-19 patients, female participants scored lower in role limitations due to emotional issues, energy/fatigue, pain, and emotional well-being. In COVID-19 patients, older age was associated with lower physical functioning, and past psychiatric history was linked to poorer functioning in all SF-36 domains except physical functioning.

### Physical and Psychosocial Well-Being of the General Public Amid the COVID-19 Pandemic

When compared to the normative data of the Arabic version of SF-36, the general population group had SF-36 scores between one standard deviation below the mean and the mean (17). Most of the previous studies that examined the impact of the pandemic on the general population did not directly assess the HRQoL but rather “assumed” that the HRQoL was affected because they found a high prevalence of depressive, anxiety, and stress symptoms (22, 23). For example, one Chilean study that directly assessed the HRQoL in the general population found the HRQoL to be affected in 1,082 adults, between 18 and 60 years old, who were quarantined by the COVID-19 health alert but who were neither confirmed nor suspected cases of COVID-19 (9). Similarly, in a group of Italian non-infected women aged between 28 and 50, SF-36 scores were significantly decreased (10). However, a Dutch study did not find the level of mental well-being during the peak of COVID-19 to be lower than in 2018 (24). This discrepancy could be due to the differences in participant selection criteria, the times the studies took place regarding the pandemic course, and the variations in COVID-19-related restrictions from country to country throughout the pandemic. Overall, there seems to be a paucity of data regarding the potential impact of the pandemic on the general population's HRQoL, even though most of the available indirect evidence suggests a likely negative impact (1, 23).

### Physical and Psychosocial Well-Being of Patients With COVID-19

The results of our study showing that COVID-19 patients might not have a poorer HRQoL than controls might be unexpected. Indeed, a growing number of studies showed a high prevalence of depression, anxiety, stress, and insomnia symptoms in patients with COVID-19 (2, 3). Based on these findings, it is often “assumed” that COVID-19 infection negatively affects physical and psychosocial well-being. However, most of these studies lacked a control group, and only a few directly examined the impact of the disease on the patient's quality of life and functioning. Thus, one can argue that while the prevalence of depressive, anxiety, and stress symptoms are high in COVID-19 patients, these can also increase in the general population amid the pandemic (1, 25). In addition, studies directly comparing infected to non-infected individuals found that infected ones had more pronounced depressive and anxiety symptoms (26), including one study from Qatar (27), but this was not the case for other studies (23).

Moreover, recent studies suggested that the prevalence of depressive and anxiety symptoms in COVID-19 patients is overestimated due to possible overlaps between these symptoms and certain features of the COVID-19 infection, including fatigue, loss of appetite, sleep disturbance, pain, and palpitations (28). In this regard, studies using the Hospital Anxiety and Depression Scale (HADS) (29), designed to identify emotional symptoms of depression in patients with concurrent somatic illness, reported a lower prevalence of depression than those using the Patient Health Questionnaire (PHQ-9), designed to cover all bodily and emotional features (2). Thus, it seems likely that the high prevalence of depression, anxiety, and stress has affected the population as a whole, rather than COVID-19 infected patients in particular.

It is hypothesized that COVID-19 infected patients might have depressive and anxiety symptoms due to the virus's potential neurotropic effects, the immune response, and the isolation due to hospitalization or quarantine (2, 30). However, the biological effects of the virus on the brain are not possibly of clinical significance in most patients. Previous studies, including a meta-analysis of longitudinal studies and natural quarantine experiments, have not found that quarantine had any major impact on mental health (31, 32). It is also possible that infected individuals have benefited from more support from their families, friends, and frequent mental health screening or interventions (23).

In addition, our samples had diverse sociocultural backgrounds, and the distribution of nationalities among the groups was different. Such cultural variation might have impacted the SF-36 scores since the expression of emotions and tendency toward somatization can greatly differ from culture to culture (33).

The mean SF-36 scores for physical and emotional components in our study were comparable to the scores reported in other studies (4–7). Out of these four studies, two interpreted the HRQoL in COVID-19 as being “low.” However, the patients' Warwick-Edinburgh Mental Well-being Scales (WEMWBS) did not differ from the population norms (6). Chen et al. (7) findings were even closer to ours: they found that one month after discharge, COVID-19 patients scored lower than the Chinese population norm only in certain HRQoL domains (social functioning and role limitations due to physical and emotional problems). However, they scored higher on other domains (mental health, bodily pain, vitality, general health) with no difference for physical functioning compared to population norms (7).

These findings, including ours, suggest that while the HRQoL in COVID-19 patients is probably affected, it is not necessarily more so than the general population amid the pandemic. In this context, the HRQoL was found to be less affected in COVID-19 patients than in their family members (34). Furthermore, the anticipation of the infection might cause more psychological distress than the infection itself since worrying about a negative event is often more anxiogenic than the occurrence of the event itself (35).

Even though most assume that hospitalized COVID-19 patients may experience higher rates of depression, anxiety, and stress, than non-hospitalized patients, other findings did confirm this. Indeed, previous studies reported the prevalence of depressive, anxiety, and PTSD symptoms in never-hospitalized COVID patients to be similar or even higher than in hospitalized patients (De (3, 36–39)). It is possible that being hospitalized in a protective environment helped to reassure the patients. COVID-19 inpatients reported medical staff care as the main supportive factor as it gave them “a sense of security” (40). Hospitalized COVID-19 patients may also have been more commonly screened for mental health issues and benefited from mental health services during their stay (39). In addition, going through the experience of a potentially severe illness and recovering from it can make people cherish the good aspects of their life, resulting in a positively biased perception of their HRQoL. Such an initial “euphoria” has been reported in patients who survived critical medical conditions (41) and in Ebola survivors (42). In a study examining the psychological experience of COVID-19 patients during a hospital stay, most patients endorsed how the thought that their lives could have suddenly ended made them realize how valuable their life, their families, and their friends are. In a sense, surviving COVID-19 can change perspectives and enhance psychological growth (40).

### Factors Associated With Poorer Physical and Psychosocial Well-Being in Patients With COVID-19

Our results suggested that female COVID-19 patients may have poorer HRQoL than males, particularly in role limitations due to emotional problems, fatigue, pain, and emotional well-being. These findings align with previous studies among COVID-19 patients showing that the prevalence of depression, anxiety, stress, and insomnia symptoms were higher in women than men (2, 3). Similarly, Chen et al. reported that the female gender was associated with poorer physical functioning, bodily pain, and role limitations due to emotional problems (2, 3). Furthermore, a similar gender difference is reported at the general population level in most studies using the SF-36 in different countries (43–45), including Arab countries (19, 46, 47).

We also found that older age in infected patients was associated with lower physical functioning. This association was also reported previously (7). It was attributed to the poor prognosis of COVID-19 in the elderly (48) and the physiological decline of physical functioning with age in the general population (44, 45). In our study, we did not find age to be associated with mental health-related HRQoL. Associations between older age and mental health in COVID-19 patients have been inconsistent, with some studies reporting better (49) and others reporting worse outcomes in the elderly (3).

In the present study, past psychiatric history in COVID-19 patients was linked to poorer functioning in all SF-36 domains except physical functioning. This link is expected given that COVID-19 patients with prior psychiatric history have been reported to experience higher levels of anxiety, depression, stress, and sleep disturbance than patients with no psychiatric history (3). In addition, patients with mental illness showed an increased risk of contracting COVID-19 and higher hospitalization rates and death compared to individuals with no history of mental illness (50).

### Strengths and Limitations

The present study is one of the few studies to focus on mental health outcomes in patients with COVID-19 infection. Furthermore, contrary to most other studies about mental health consequences of the COVID-19 disease, which merely examined the prevalence of anxiety, depression, and stress symptoms, the present study scrutinized different domains of psychosocial and physical well-being (3, 25).

However, some limitations need to be acknowledged. For example, although we used MANCOVA to control the differences in certain sociodemographic characteristics between the three groups, these variations could still bias the proper comparisons. Moreover, mental health issues or the poor perceived HRQoL may have affected the decision to participate in the survey, especially among controls. This bias might have caused controls to score poorer than expected. Social-desirability bias might also have influenced certain participant's answers (51). Besides, we could not capture certain variables that may have affected HR-QoL (severity of COVID-19 beyond the mere need for hospitalization, duration of hospitalization, and the exact time elapsed between discharge and filling the questionnaire). In addition, like most previous studies, the cross-sectional design of the present study does not allow to distinguish between short-term and long-term effects on well-being in COVID-19 patients. A prospective design could have helped disentangle the acute consequences of the infection from any potential long-term sequelae (2, 3).

## Data Availability Statement

The raw data supporting the conclusions of this article will be made available upon request from the corresponding author.

## Ethics Statement

The studies involving human participants were reviewed and approved by IRB of Hamad Medical Corporation. Written informed consent for participation was not required for this study in accordance with the national legislation and the institutional requirements.

## Author Contributions

SG, HA-A, and NH formalized the research concept and proposal. ZA-A supported with literature review, administrative aspects of the research, randomized, and distributed subjects. NH, FK, AA, PD, ABW, MT, and MK conducted the survey and entered the data. SO did the statistical analysis and wrote the manuscript. SG and HA-A reviewed and edited the manuscript. SG was responsible for the overall project and supervised all steps associated with the study as the lead principal investigator. MAM supported the project by providing data on patients admitted to either hospital or quarantine facilities. All authors contributed to the article and approved the submitted version.

## Conflict of Interest

The authors declare that the research was conducted in the absence of any commercial or financial relationships that could be construed as a potential conflict of interest.

## Publisher's Note

All claims expressed in this article are solely those of the authors and do not necessarily represent those of their affiliated organizations, or those of the publisher, the editors and the reviewers. Any product that may be evaluated in this article, or claim that may be made by its manufacturer, is not guaranteed or endorsed by the publisher.
